# Choline restores respiration in Psd1-deficient yeast by replenishing mitochondrial phosphatidylethanolamine

**DOI:** 10.1016/j.jbc.2021.100539

**Published:** 2021-03-12

**Authors:** Donna M. Iadarola, Alaumy Joshi, Cameron B. Caldwell, Vishal M. Gohil

**Affiliations:** Department of Biochemistry and Biophysics, MS 3474, Texas A&M University, College Station, Texas, USA

**Keywords:** phospholipid, mitochondria, phosphatidylethanolamine, yeast, choline, ethanolamine, Psd1, Psd2, Vps39, CDP-Etn, CDP–ethanolamine, Cho, choline, CL, cardiolipin, ER, endoplasmic reticulum, Etn, ethanolamine, MRC, mitochondrial respiratory chain, PC, phosphatidylcholine, PE, phosphatidylethanolamine, PS, phosphatidylserine, SC, synthetic complete

## Abstract

Phosphatidylethanolamine (PE) is essential for mitochondrial respiration in yeast, *Saccharomyces cerevisiae*, whereas the most abundant mitochondrial phospholipid, phosphatidylcholine (PC), is largely dispensable. Surprisingly, choline (Cho), which is a biosynthetic precursor of PC, has been shown to rescue the respiratory growth of mitochondrial PE-deficient yeast; however, the mechanism underlying this rescue has remained unknown. Using a combination of yeast genetics, lipid biochemistry, and cell biological approaches, we uncover the mechanism by showing that Cho rescues mitochondrial respiration by partially replenishing mitochondrial PE levels in yeast cells lacking the mitochondrial PE-biosynthetic enzyme Psd1. This rescue is dependent on the conversion of Cho to PC *via* the Kennedy pathway as well as on Psd2, an enzyme catalyzing PE biosynthesis in the endosome. Metabolic labeling experiments reveal that in the absence of exogenously supplied Cho, PE biosynthesized *via* Psd2 is mostly directed to the methylation pathway for PC biosynthesis and is unavailable for replenishing mitochondrial PE in Psd1-deleted cells. In this setting, stimulating the Kennedy pathway for PC biosynthesis by Cho spares Psd2-synthesized PE from the methylation pathway and redirects it to the mitochondria. Cho-mediated elevation in mitochondrial PE is dependent on Vps39, which has been recently implicated in PE trafficking to the mitochondria. Accordingly, epistasis experiments placed Vps39 downstream of Psd2 in Cho-based rescue. Our work, thus, provides a mechanism of Cho-based rescue of mitochondrial PE deficiency and uncovers an intricate interorganelle phospholipid regulatory network that maintains mitochondrial PE homeostasis.

Mitochondrial membranes have a highly conserved and unique phospholipid composition, characterized by high abundance of nonbilayer phospholipids such as phosphatidylethanolamine (PE) and cardiolipin (CL) (1, 2). Perturbation of this phospholipid composition alters the bulk physical properties of the membrane and specific phospholipid–protein interactions, disrupting many processes including but not limited to mitochondrial bioenergetics, cristae architecture, and protein import (1, 3, 4). A systematic analysis of the role of phospholipids in mitochondrial bioenergetics has uncovered specific requirements of PE and CL in the assembly and activity of mitochondrial respiratory chain (MRC) complexes (5). Our recent study has also uncovered a specific requirement of CL in the stability and function of the mitochondrial calcium channel (6). These studies show that mitochondrial function primarily depends on the nonbilayer phospholipids, and within this class of phospholipids, individual nonbilayer phospholipids, PE and CL, perform specific roles.

Among all the major classes of phospholipids, PE is the second most abundant mitochondrial phospholipid and is essential for respiration in yeast and mammalian cells (5, 7, 8, 9). PE can be biosynthesized by multiple pathways in yeast *Saccharomyces cerevisiae* (Fig. 1). Decarboxylation of phosphatidylserine (PS) by Psd1 in the mitochondria is the major source of *in situ* and cellular PE (10, 11, 12). CDP–ethanolamine (CDP–Etn) Kennedy pathway biosynthesis of PE involves conversion of the substrate, ethanolamine (Etn), to PE by the sequential action of the enzymes Eki1/Cki1, Ect1, and Ept1/Cpt1 in the endoplasmic reticulum (ER) (13, 14, 15). In addition, decarboxylation of PS by Psd2 in the endosomal compartments generates PE (16, 17). A majority of PE biosynthesized by Psd2 is methylated to become phosphatidylcholine (PC) by methyltransferases Pem1 and Pem2 in the ER (12, 18, 19). PC can also be synthesized from choline (Cho) through the CDP–Cho Kennedy Pathway *via* the sequential action of Cki1/Eki1, Pct1, and Cpt1/Ept1 (Fig. 1) (13, 14, 15).

**Figure 1:**
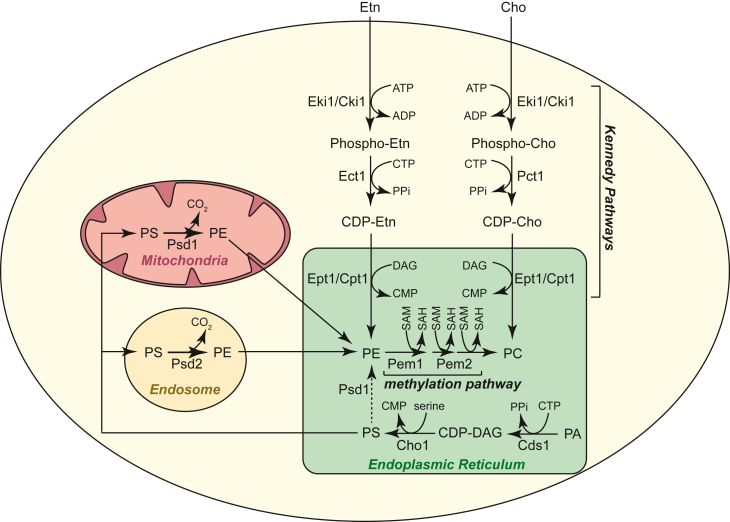
**Aminoglycerophospholipid biosynthetic pathways in yeast, *Saccharomyces cerevisiae.*** The three major pathways for PE biosynthesis are (1) mitochondrial decarboxylation of PS by Psd1, (2) endosomal decarboxylation of PS by Psd2, and (3) incorporation of Etn *via* the CDP–Etn Kennedy pathway. The two major pathways for PC biosynthesis are (1) methylation of PE by Pem1 and Pem2 and (2) incorporation of Cho *via* the CDP–Cho Kennedy pathway. Phosphatidylserine is biosynthesized from PA in a two-step reaction involving CDP–DAG synthesis followed by the addition of serine by Cho1. ADP, adenosine diphosphate; ATP, adenosine triphosphate; CDP, cytidine diphosphate; Cho, choline; CMP, cytidine monophosphate; CTP, cytidine triphosphate; DAG, diacylglycerol; Etn, ethanolamine; PA, phosphatidic acid; PC, phosphatidylcholine; PE, phosphatidylethanolamine; PPi, pyrophosphate; PS, phosphatidylserine; SAH, S-adenosylhomocysteine; SAM, S-adenosylmethionine.

Unlike PE, depletion of PC, the most abundant bilayer-forming mitochondrial phospholipid, does not impair MRC function or formation in yeast (5, 20). The respiratory defect of *psd1Δ* cells is specifically due to a decrease in PE-dependent activities of MRC complexes III and IV (5, 8). Notably, stimulating the CDP–Etn Kennedy pathway of PE biosynthesis by Etn supplementation can restore respiratory growth and mitochondrial respiration of *psd1Δ* cells (5, 21). The Etn-mediated rescue requires transport of PE from the ER to the mitochondria in a Vps39-dependent manner (22). This rescue of mitochondrial PE deficiency by Etn supplementation is consistent with the critical requirements of nonbilayer phospholipids for mitochondrial functions (23, 24). However, rescue of respiratory growth of *psd1Δ* cells by Cho (21) is surprising, considering that Cho is a precursor for the bilayer-forming PC (Fig. 1).

Here, we uncover the mechanism of Cho-based rescue of mitochondrial respiration in *psd1Δ* cells. We show that Cho supplementation stimulates PC biosynthesis *via* the CDP–Cho Kennedy pathway, alleviating the need for PC biosynthesis from PE *via* the methylation pathway. The PE, biosynthesized *via* Psd2, is thus spared from entering the methylation pathway and becomes available for mitochondrial function. Apart from CDP–Cho Kennedy pathway enzymes and Psd2, the Cho-based rescue of mitochondrial PE deficiency also requires Vps39, implicating its role in the mitochondrial PE transport. These findings reinforce the idea that the MRC function specifically requires nonbilayer phospholipids.

## Results

### Pct1 and Psd2 are essential for Cho-mediated rescue of psd1Δ respiratory growth

**Figure 2:**
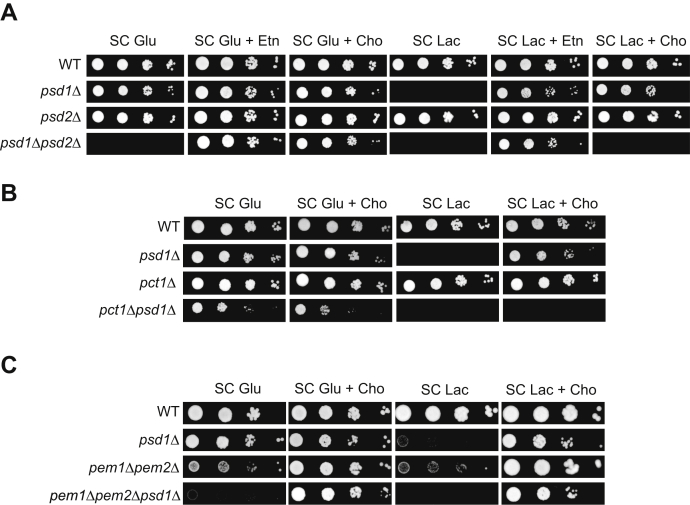
**Psd2 and Pct1 are essential for Cho-mediated respiratory growth rescue of *psd1Δ* cells.** Ten-fold serial dilution of the indicated yeast mutants (*A*–*C*) was seeded onto synthetic complete (SC) glucose and SC lactate plates with and without Etn or Cho supplementation. Images were captured after 2 days (SC glucose media) and 5 days (SC lactate media) of growth at 30 °C. The figures are representative images from three independent biological replicates. Cho, choline; Etn, ethanolamine.

Consistent with the previous study (21), we found that Cho supplementation can rescue growth of *psd1Δ* cells in the respiratory (synthetic complete [SC] Lac) medium but can only rescue growth of *psd1Δpsd2Δ* cells in the fermentative (SC Glu) medium (Fig. 2*A*). These results indicate that PE biosynthesis *via* Psd2 is essential for Cho-based rescue of *psd1Δ* cells in respiratory growth conditions. Next, we asked if PC biosynthesis *via* the CDP–Cho Kennedy pathway is essential for Cho-mediated respiratory growth rescue of *psd1Δ* cells by deleting Pct1, an essential enzyme of the CDP–Cho pathway (Fig. 1). Pct1-deleted cells displayed normal growth in fermentable or respiratory media (Fig. 2*B*); however, deletion of Pct1 in a *psd1Δ* background abrogated Cho-mediated rescue of respiratory growth (Fig. 2*B*). Taken together, these results show that Psd2 and Pct1 are both essential for Cho-mediated rescue of *psd1Δ* cells.

Because Psd2 provides endosomal PE as a substrate for PC biosynthesis *via* the methylation pathway (12) (Fig. 1), we wondered whether this PC is essential for *psd1Δ* rescue. To test this idea, we used the *pem1Δpem2Δpsd1Δ* strain where Pem1 and Pem2, the PE methylation pathway enzymes, are deleted in a *psd1Δ* background. Loss of *pem1Δpem2Δ* did not abrogate Cho-mediated respiratory growth rescue of *psd1Δ* cells (Fig. 2*C*). Taken together, these results suggest that Psd2 contributes to Cho-mediated rescue of *psd1Δ* cells by providing PE to cells but not PE as a substrate for PC biosynthesis *via* the methylation pathway.

### Cho supplementation increases mitochondrial PE in psd1Δ cells

**Figure 3:**
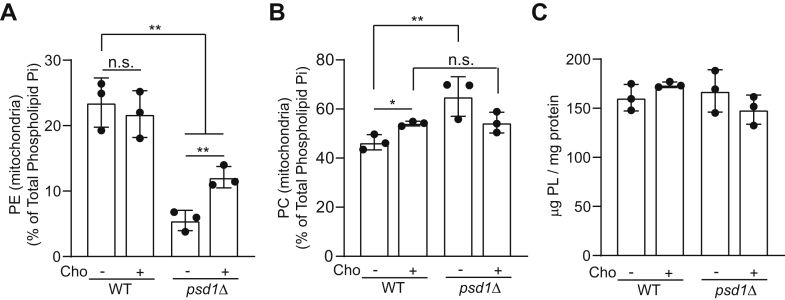
**Choline supplementation increases mitochondrial PE levels in *psd1Δ* cells.***A*, PE levels of density gradient–purified mitochondria from WT and *psd1Δ* cells cultured in SC lactate ± Cho media. *B*, PC levels of density gradient–purified mitochondria from WT and *psd1Δ* cells cultured in SC lactate ± Cho media. *C*, the total phospholipid content of density gradient–purified mitochondria from SC lactate ± Cho grown cells. PE and PC levels are expressed as the percent of total phospholipid phosphorous. Data are expressed as the mean ± SD; ∗*p* < 0.05, ∗∗*p* < 0.01, (n = 3). Each data point represents a biological replicate. Cho, choline; n.s., not significant; PC, phosphatidylcholine; PE, phosphatidylethanolamine; SC, synthetic complete.

To understand how supplementing Cho, which feeds into PC biosynthesis, could rescue the respiratory growth of *psd1Δ* cells, we measured the steady state levels of mitochondrial phospholipids. Unexpectedly, we found that Cho supplementation significantly increased mitochondrial PE levels in *psd1Δ* cells (Fig. 3*A*). Furthermore, Cho supplementation also normalized mitochondrial PC levels, which are elevated in *psd1Δ* cells (Fig. 3*B*). The increased levels of mitochondrial PE upon Cho supplementation are similar to those we had previously observed with Etn supplementation (5) and can explain the respiratory growth rescue of *psd1Δ* cells. We considered the possibility that Cho supplementation may increase the overall abundance of mitochondrial phospholipids, which may explain the respiratory growth rescue of *psd1Δ* cells. Upon measuring the absolute phospholipid levels per mg of mitochondrial protein, we found that the overall abundance of mitochondrial phospholipids is unaffected in WT and *psd1Δ* cells with or without Cho supplementation (Fig. 3*C*). From these results, we infer that the absolute phospholipid levels do not contribute to Cho-mediated rescue but rather that elevated PE is the most likely reason for the rescue.

### Cho supplementation restores mitochondrial respiration in psd1Δ cells

**Figure 4:**
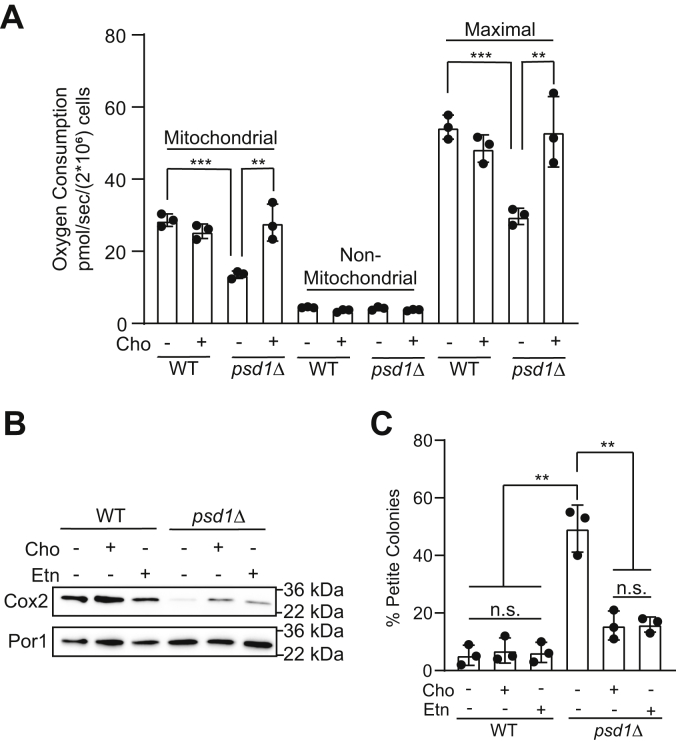
**Choline supplementation rescues mitochondrial bioenergetics, Cox2 levels, and petite formation in *psd1Δ* cells.***A*, the rate of oxygen consumption was measured from WT and *psd1Δ* cells cultured in the SC lactate ± Cho media. Maximal respiration refers to respiration after addition of CCCP, and non-mitochondrial respiration refers to antimycin-resistant respiration. *B*, SDS-PAGE/Western blot analysis of Cox2 levels from mitochondria isolated from WT and *psd1Δ* cells cultured in the SC glucose media with or without Etn or Cho supplementation. Por1, a mitochondrial outer membrane protein, is used as a loading control. *C*, the percentage of respiratory growth–deficient petite colonies of WT and *psd1Δ* cells cultured in SC glucose media with or without Etn or Cho supplementation. Data are expressed as the mean ± SD; ∗∗*p* < 0.01, ∗∗∗*p* < 0.001 (n = 3). The Western blot image is a representative of three independent experiments, and each data point on the *bar charts* represents a biological replicate. CCCP, carbonyl cyanide m-chlorophenyl hydrazone; Cho, choline; Etn, ethanolamine; n.s., not significant; SC, synthetic complete.

The severely compromised respiratory growth of *psd1Δ* cells is attributed to reduced mitochondrial respiration (5, 25). Indeed, we have previously shown that partially replenishing mitochondrial PE levels by Etn supplementation can rescue respiratory growth of *psd1Δ* cells by restoring mitochondrial respiration (5). Therefore, we asked whether elevating mitochondrial PE levels by Cho supplementation can also restore mitochondrial respiration and found that Cho supplementation restored both basal and uncoupler-stimulated maximal respiration in *psd1Δ* cells (Fig. 4*A*). To investigate the biochemical basis of this rescue, we measured the steady state levels of Cox2, a mitochondrial DNA–encoded subunit of the MRC complex IV, which is known to be decreased in glucose-grown *psd1Δ* cells (5). Consistent with the partial restoration of mitochondrial PE levels, Cho supplementation also partially rescued Cox2 levels in *psd1Δ* cells (Fig. 4*B*). The decrease in Cox2 levels has been attributed to the frequent loss of mitochondrial DNA in *psd1Δ* cells, which results in the formation of petite colonies (5, 10). Therefore, we measured the frequency of petite formation in *psd1Δ* cells and found that Cho supplementation significantly reduced petite formation (Fig. 4*C*). These data provide the biochemical basis of Cho-mediated rescue of the respiratory growth of *psd1Δ* cells.

### Cho supplementation reduces PE to PC conversion *via* the methylation pathway

**Figure 5:**
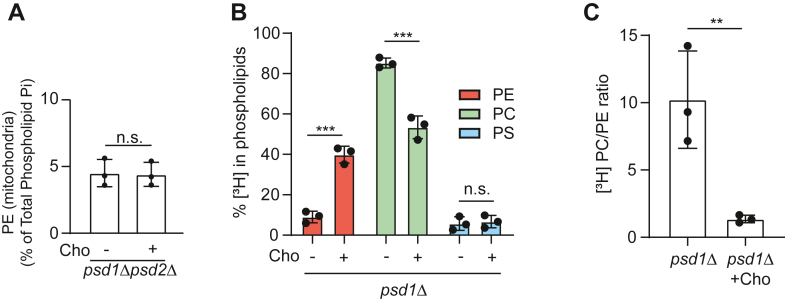
**PE to PC conversion is reduced upon Cho supplementation.***A*, PE levels of density gradient–purified mitochondria from *psd1Δpsd2Δ* cells cultured in SC glucose ± Cho media. PE levels are expressed as the percent of total phospholipid phosphorous. *B*, the percent of [^3^H] PS, PE, and PC from *psd1Δ* cells grown in the SC glucose ± Cho media containing 10-μCi [^3^H] serine. *C*, the ratio of [^3^H] PC/PE from *psd1Δ* cells grown in the SC glucose ± Cho media containing 10-μCi [^3^H] serine. Panels *B* and *C* are data from the same experiment. Data are expressed as the mean ± SD; ∗∗*p* < 0.01, ∗∗∗*p* < 0.001 (n = 3). Each data point represents a biological replicate. n.s., not significant; PC, phosphatidylcholine; PE, phosphatidylethanolamine; PS, phosphatidylserine; SC, synthetic complete.

Next, we focused on understanding the mechanism underlying the Cho-based increase in mitochondrial PE levels. Upon steady state phospholipid measurement of purified mitochondria, we found that unlike in *psd1Δ* cells, Cho supplementation did not increase mitochondrial PE levels in *psd1Δpsd2Δ* cells, implying that Psd2-derived PE is responsible for elevating mitochondrial PE (Fig. 5*A*). To understand how Cho supplementation impacts Psd2-catalyzed PE biosynthesis, we performed a radiolabeling experiment using [^3^H] serine, which enters the phospholipid biosynthetic pathway by incorporation into PS, the substrate of Psd2 (Fig. 1). [^3^H] serine labeling in the presence of Cho supplementation led to an increased accumulation of the radiolabel in PE at the expense of PC (Fig. 5*B*), which resulted in a ∼10-fold decrease in the radiolabeled PC/PE ratio (Fig. 5*C*). These results suggest that the contribution of Psd2-synthesized PE to PC is decreased upon Cho supplementation, and this spared PE can be diverted to replenish mitochondrial PE in *psd1Δ* cells.

### Vps39 is essential for Cho-mediated rescue of psd1Δ cells

**Figure 6:**
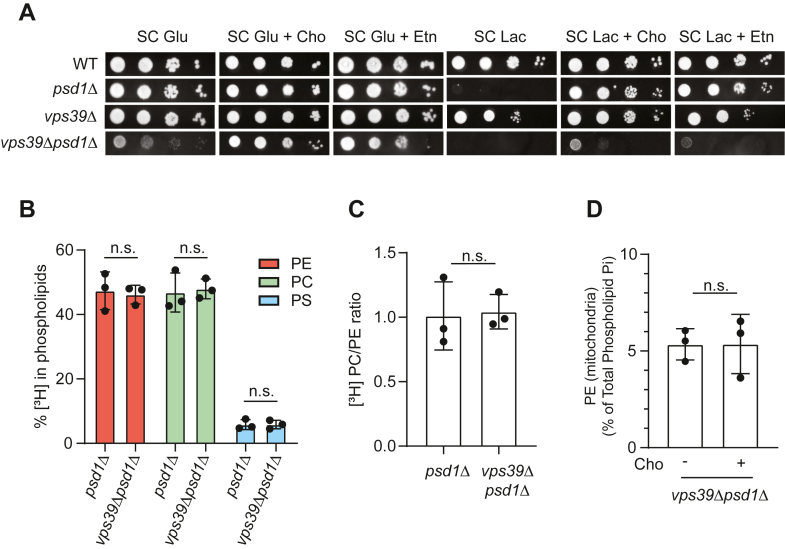
**Vps39 is essential for Cho-mediated elevation in mitochondrial PE levels in *psd1Δ* cells.***A*, ten-fold serial dilution of the indicated yeast mutants seeded onto the indicated agar plates. Images were captured after 2 days of growth in the SC glucose media and 5 days of growth in the SC lactate media. The images are representative of three independent biological replicates. *B*, the percent of [^3^H] PS, PE, and PC in *psd1Δ* and *vps39Δpsd1Δ* cells grown in the SC glucose media supplemented with Cho. *C*, the ratio of [^3^H] PC/PE from *psd1Δ* and *vps39Δpsd1Δ* cells grown in SC glucose + Cho. *D*, PE levels of density gradient–purified mitochondria isolated from *vps39Δpsd1Δ* cells cultured in SC lactate ± Cho. Data are expressed as the mean ± SD; n = 3. Each data point represents a biological replicate. Cho, choline; n.s., not significant; PC, phosphatidylcholine; PE, phosphatidylethanolamine; PS, phosphatidylserine; SC, synthetic complete.

We have recently shown that Vps39 is required for Etn-mediated elevation in mitochondrial PE levels (22). Therefore, we wondered whether Cho-mediated elevation of mitochondrial PE in *psd1Δ* cells also requires Vps39. Consistent with the requirement of Vps39 in Etn-mediated rescue of the respiratory growth of *psd1Δ* cells, we find that Cho-mediated respiratory growth rescue of *psd1Δ* cells also required Vps39 (Fig. 6*A*). To determine if Vps39 acts upstream or downstream of Psd2 in Cho-mediated rescue, we used the [^3^H] serine–radiolabeling strategy to determine if the loss of Vps39 perturbs PS, PE, and PC biosynthesis in *psd1Δ* cells. If Vps39 acts upstream of Psd2, then we expect decreased incorporation of radiolabeled serine in PE and PC. If Vps39 acts downstream of Psd2, then there will be no change in Psd2 activity and the incorporation of radiolabel in PS, PE, and PC will be identical to Cho-supplemented *psd1Δ* cells. Consistent with this later model, we find that the percent of radiolabeled PS, PE, and PC is the same for *psd1Δ* and *vps39Δpsd1Δ* cells (Fig. 6*B*), and the ratio of PC/PE is unchanged between these two strains (Fig. 6*C*).

To directly test the requirement of Vps39 in Cho-mediated PE elevation in *psd1Δ* mitochondria (Fig. 3*A*), we measured the steady state levels of mitochondrial PE in *vps39Δpsd1Δ* cells with and without Cho supplementation and found that PE levels are not elevated upon Cho supplementation (Fig. 6*D*). Taken together, these results show that loss of Vps39 does not impair PE biosynthesis *via* Psd2 but specifically abrogates the delivery of Psd2-synthesized PE to *psd1Δ* mitochondria.

## Discussion

In this study, we sought to determine the mechanism of Cho-mediated rescue of mitochondrial PE deficiency and found that Cho supplementation restores mitochondrial bioenergetic functions in PE-deficient *psd1Δ* cells by elevating mitochondrial PE levels through a “lipid-sparing” mechanism. Our study has uncovered a novel intracellular phospholipid homeostatic mechanism that maintains the mitochondrial requirements of bilayer and nonbilayer phospholipids *via* cross-pathway regulation of the Kennedy and methylation pathways of PC biosynthesis.

**Figure 7:**
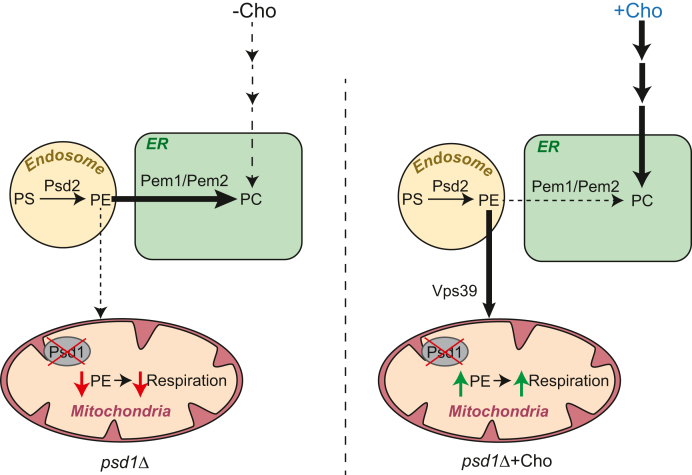
**A model depicting Cho-mediated restoration of mitochondrial PE levels in Psd1-deficient mitochondria.** The *left panel* shows that in the absence of exogenous supplementation of Cho, the majority of endosomal PE is utilized for PC biosynthesis *via* the Pem1/Pem2-methylation pathway and the contribution of endosomal PE to the mitochondria is low. In this scenario, the mitochondrial PE levels remain low, which results in reduced respiration. The panel to the *right* shows that Cho supplementation stimulates PC biosynthesis *via* the Kennedy pathway, which diverts endosomal PE to the mitochondria in a Vps39-dependent manner, elevating mitochondrial PE levels and restoring respiration. Cho, choline; PE, phosphatidylethanolamine.

Redundant pathways of PE and PC biosynthesis exist in *S. cerevisiae*; however, how perturbation in one pathway impacts the flux through the other pathway to maintain homeostatic balance is not fully understood (26, 27). By analyzing the incorporation of radiolabeled serine into aminoglycerophospholipids—PS, PE, and PC—in mitochondrial PE-deficient *psd1Δ* cells, we found that Cho-mediated stimulation of PC synthesis *via* the Kennedy pathway obviates the need for PC biosynthesis from the PE methylation pathway. The “spared” endosomal PE can instead be diverted to the mitochondria to compensate for the lack of mitochondrial PE biosynthesis in cells lacking Psd1 (Fig. 7). This observation raises an important question: what is the molecular mechanism that reduces PE to PC conversion *via* the methylation pathway upon Cho supplementation? The essential requirement of Pct1 for Cho-mediated rescue of *psd1Δ* cells (Fig. 2*B*) suggests that PC synthesis from Cho *via* the Kennedy pathway is necessary for reducing PE to PC conversion by the methylation pathway. Thus, a possible mechanism by which Cho-derived PC could reduce the flux through the methylation pathway is by inhibiting the methylation pathway enzymes, as has been suggested previously (28).

The requirement of Psd2 for Cho-based elevation of mitochondrial PE (Fig. 5*A*) implies that PE biosynthesized in the endosomal compartment by Psd2 is transported to the mitochondria. Therefore, we wanted to identify the molecular player(s) required for this trafficking of PE. Our recent study has implicated Vps39 in PE trafficking from the ER to the mitochondria (22). So, we asked whether Vps39 also plays a role in trafficking Psd2-synthesized PE to the mitochondria. We found that Vps39 is indeed essential for the Cho-mediated elevation of mitochondrial PE levels in *psd1Δ* cells (Fig. 6*D*). Importantly, radiolabeling experiments place Vps39 downstream of Psd2 (Fig. 6*B*), thus eliminating its role in PE biosynthesis. Although currently we do not know whether Psd2-synthesized PE is transported directly from the endosomes or through the ER, the results from this study emphasize a broader role of Vps39 in the trafficking of both the Kennedy pathway–synthesized PE and Psd2-synthesized PE to the mitochondria.

One of the important implications of our finding is the possibility of using Cho supplementation as a means to ameliorate mitochondrial dysfunction in human patients with mutations in mitochondrial PISD, a human homolog of yeast Psd1 (7, 29, 30). Because humans do not have the Psd2 equivalent pathway (31), the same Psd2-dependent restoration of mitochondrial PE by Cho supplementation is unlikely. However, in mammals, as in yeast, a significant fraction of PE is used up in the biosynthesis of PC *via* the methylation pathway in hepatocytes (31), which can exacerbate PE deficiency in the liver of human patients with partial loss-of-function mutations in PISD (29). Thus, stimulating PC biosynthesis *via* Cho supplementation in these patients could spare mitochondrial PE for respiratory function and may ameliorate disease symptoms. Thus, the presence of multiple pathways of PE and PC biosynthesis in yeast and humans could allow for a lipid-sparing mechanism necessary for Cho-based rescue.

In summary, our study has uncovered a mechanism by which Cho supplementation can restore mitochondrial function in *psd1Δ* cells by directing endosomal PE to the mitochondria. This finding further emphasizes the critical requirements of nonbilayer phospholipids for mitochondrial functions.

## Experimental procedures

### Yeast strains, growth media composition, and culture conditions

All *S. cerevisiae* strains used in this study are listed in Table 1. Yeast cells were maintained and precultured in YPGE media (1% Yeast extract, 2% Peptone, 3% Glycerol, and 1% Ethanol) or YPD medium (1% Yeast extract, 2% Peptone, and 2% Glucose) for strains that cannot grow in the YPGE medium. For final culture, yeast were grown in the SC medium (0.17% yeast nitrogen base without amino acids, 0.5% ammonium sulfate, 0.2% dropout mix containing amino acids) with either glucose (2%) or lactate (2% and pH 5.5) as a carbon source (32). Etn, Cho, or serine (Ser) were added at 2-mM concentrations to the SC media, wherever indicated. Cultures were started at an absorbance at 600 nm of 0.1 and were grown to the late logarithmic phase at 30 °C. The solid medium was prepared with the addition of 2% agar. Growth measurements on agar plates were performed by seeding 3 μl of 10-fold serial dilutions of preculture onto the indicated plates. The pictures were taken after 2 days of growth in the SC glucose medium and 5 days of growth in the SC lactate medium. The petite formation assay was performed by spreading ∼200 cells from the SC glucose medium onto YPD and YPGE plates. Percent petite colonies were calculated by counting the number of colonies grown in YPGE and YPD. KO yeast strains were constructed by one-step gene disruption using geneticin, hygromycin, and nourseothricin cassettes (33).

**Table 1:** *Saccharomyces cerevisiae* strains used in this study

Yeast strains	Genotype	Source
BY4741 WT	*MATa, his3Δ1, leu2Δ0, met15Δ0, ura3Δ0*	Miriam L. Greenberg
BY4741 *psd1Δ*	*MATa, his3Δ1, leu2Δ0, met15Δ0, ura3Δ0, psd1Δ::hphNT1*	This study
BY4741 *pct1Δ*	*MATa, his3Δ1, leu2Δ0, met15Δ0, ura3Δ0, pct1Δ::kanMX4*	Open Biosystems
BY4741 *psd2Δ*	*MATa, his3Δ1, leu2Δ0, met15Δ0, ura3Δ0, psd2Δ::kanMX4*	Open Biosystems
BY4741 *psd1Δ psd2Δ*	*MATa, his3Δ1, leu2Δ0, met15Δ0, ura3Δ0, psd2Δ::kanMX4, psd1Δ::hphNT1*	This study
BY4741 *pct1Δ psd1Δ*	*MATa, his3Δ1, leu2Δ0, met15Δ0, ura3Δ0, pct1Δ::kanMX4, psd1Δ::hphNT1*	This study
W3031A WT	*MATa, ade2-1, ura3-1, his3-11,15, trp1-1, leu2-3,112, can1-100*	Akinori Ohta
SKY010 *pem1Δ pem2Δ*	*MATa, ade2-1, ura3-1, his3-11,15, trp1-1, leu2-3,112, can1-100, pem1Δ::HIS3, pem2Δ::hph*	Akinori Ohta
SKY011 *pem1Δ pem2Δ psd1Δ*	*MATa, ade2-1, ura3-1, his3-11,15, trp1-1, leu2-3,112, can1-100, pem1Δ::HIS3, pem2Δ::hph, psd1Δ::ADE2*	Akinori Ohta
W3031A *psd1Δ*	*MATa, ade2-1, ura3-1, his3-11,15, trp1-1, leu2-3,112, can1-100, psd1Δ::hphNT1*	This study
BY4741 *vps39Δ*	*MATa, his3Δ1, leu2Δ0, met15Δ0, ura3Δ0, vps39Δ::kanMX4*	Open Biosystems
BY4741 *vps39Δpsd1Δ*	*MATa, his3Δ1, leu2Δ0, met15Δ0, ura3Δ0, vps39Δ::kanMX4, psd1Δ::hphNT1*	This study

### Mitochondria isolation

Isolation of crude and pure mitochondria was performed as described previously (22, 34). Briefly, yeast cells were spheroplasted using zymolyase digestion. Spheroplasts were lysed by homogenization using a glass Teflon homogenizer. The crude mitochondrial preparation was obtained after centrifugation steps of 1500*g*, 4000*g*, and 12,000*g*. We performed sucrose density gradient centrifugation to obtain higher purity mitochondria as described previously (34). The purity of mitochondrial isolation for these strains has been optimized as per our previous reports (5, 22).

### SDS-PAGE and immunoblotting

Mitochondrial proteins (20 μg) were separated on Mini-PROTEAN TGX 4 to 20% stain-free gels (Bio-Rad) and transferred onto polyvinylidene fluoride membranes using a Trans-Blot SD semidry transfer cell (Bio-Rad). The membrane was then probed with the following primary antibodies Cox2, 1:50,000 (110271; Abcam) and Por1, 1:50,000 (110326; Abcam). Membranes were incubated with anti-mouse secondary antibodies (1:5000) for 1 h at room temperature, and the blot was developed using the Clarity Western ECL reagent (Bio-Rad Laboratories).

### Phospholipid extraction, separation, and quantification

Phospholipids were extracted, separated, and quantified as described previously (5, 22). Briefly, lipids were extracted from spheroplasts or mitochondria with Folch solution (2:1 chloroform:methanol) (35). The extracted lipids were first washed with water followed by another wash with 1:1 water:methanol and were then dried under nitrogen gas. Dried phospholipids were resuspended in 100% chloroform and were separated using two-dimensional TLC. Phospholipid spots were scraped from TLC plates and phosphorus from each spot was quantified using the Bartlett method (36).

### Oxygen consumption rate measurement

Oxygen consumption of SC lactate-grown cells was measured using the high-resolution O2K FluoRespirometer (Oroboros) at 30 °C. Each measurement was performed on 4 × 10^6^ cells in 2.1 ml of the SC lactate media. After basal respiration was measured, maximal respiration was determined after injecting 5-μM carbonyl cyanide m-chlorophenyl hydrazone to the cells. Non-mitochondrial respiration was measured after addition of 2-μM antimycin A.

### [^3^H] serine radiolabeling

Radiolabeled serine, 10 μCi of [3-^3^H] serine (American Radiolabeled Chemicals catalog number 0246), was added to 5-ml SC glucose cultures at a starting absorbance at 600 nm of 0.1. After 14 h of growth, cells were harvested by centrifugation at 3000*g* for 5 min followed by washing with cold water. The washed cells were resuspended in 1 ml of Zymolyase buffer (50-mM Tris-SO_4_, pH 7.4, 1.2 M glycerol, 100-mM sodium thioglycolate, and 1.5-mg zymolyase) and incubated at 30 °C for 15 min with shaking. The spheroplasts were pelleted, resuspended in 250 μl of Folch solution (2:1 chloroform:methanol), and placed on a shaker. After 1 h on the shaker, 50 μl of water was added and tubes were vortexed and again placed on the shaker for 5 min. The sample was centrifuged at 1000*g* for 2 min for phase separation. The lower organic phase was transferred to a new 1.5-ml tube, and the volume was recorded. The phospholipid containing the organic phase was washed with ∼20 μl of the 1:1 methanol:water solution. The tube was vortexed and centrifuged, and the organic layer was transferred to a new tube. The wash step was repeated one more time. One-dimensional TLC was performed as described previously (22). Briefly, 5, 10, and 20 μl of the lipid extract were loaded onto the boric acid–washed TLC plate for each condition. The plate was run with a solvent system of 25:25:1.5 chloroform:methanol:ammonium hydroxide. Iodine vapors were used to detect the phospholipids. PS, PE, and PC were scraped and put into individual tubes for each condition. 1 ml of Ultima Gold Liquid Scintillation Cocktail (PerkinElmer catalog number 6013321) was added to each tube for counting using PerkinElmer Liquid Scintillation Analyzer Tri-Carb 4910TR. Each sample was counted two times. The average of the counts was used for analysis.

### Statistical analysis

GraphPad prism software was used to determine the mean ± SD values from at least three biological replicates, which are defined as independent experiments performed on three different days each starting from a different clone. Statistical analyses were performed by unpaired Student’s *t* test, and the significance is indicated by the *p*-value in each figure.

## Data availability

All data are presented in the article.

## Conflict of interest

The authors declare that they have no conflicts of interest with the contents of this article.
